# Detection of SARS-CoV-2 pneumonia: two case reports 

**DOI:** 10.1186/s13256-020-02551-1

**Published:** 2020-12-11

**Authors:** Felix S. Seibert, Daniela Toma, Frederic Bauer, Krystallenia Paniskaki, Moritz Anft, Benjamin J. Rohn, Simon Wang, Diana Racovitan, Nina Babel, Timm H. Westhoff

**Affiliations:** 1 Medical Department 1, University Hospital Marien Hospital Herne, Ruhr University Bochum, Hölkeskampring 40, 44625 Herne, Germany; 2 Center for Translational Medicine, University Hospital Marien Hospital Herne, Ruhr University Bochum, Herne, Germany

**Keywords:** COVID-19, SARS-CoV-2, Swab test

## Abstract

**Background:**

Developing therapeutic strategies for a SARS-CoV-2 infection is challenging, but first the correct diagnosis has to be made. Unspecific upper and lower respiratory tract symptoms can be misleading; hence, a nasopharyngeal swab test with a real-time reverse-transcription-polymerase chain reaction is of great importance. However, early viral clearing jeopardizes a sound diagnosis of COVID-19.

**Case presentation:**

We report on two Caucasian patients who had negative pharyngeal swab tests at the onset of SARS-CoV-2 pneumonia. In one patient, the virus was not even detectable in bronchoalveolar lavage despite typical radiomorphologic changes.

**Conclusions:**

Negative PCR findings in both the pharynx and bronchoalveolar lavage do not exclude COVID-19 pneumonia. Computed tomography is a crucial diagnostic prerequisite in this context.

## Background

The diagnosis of 2019 coronavirus disease (COVID-19) is usually based on a positive real-time reverse-transcription-polymerase chain-reaction (RT-PCR) assay for 2019 novel coronavirus (SARS-CoV-2) using a nasopharyngeal swab test. In the following, we present two cases of COVID-19 pneumonia in subjects with negative PCR tests. These cases should alert physicians that negative PCR findings do not rule out the disease in subjects with pneumonia.

## Case presentation

**Fig. 1:**
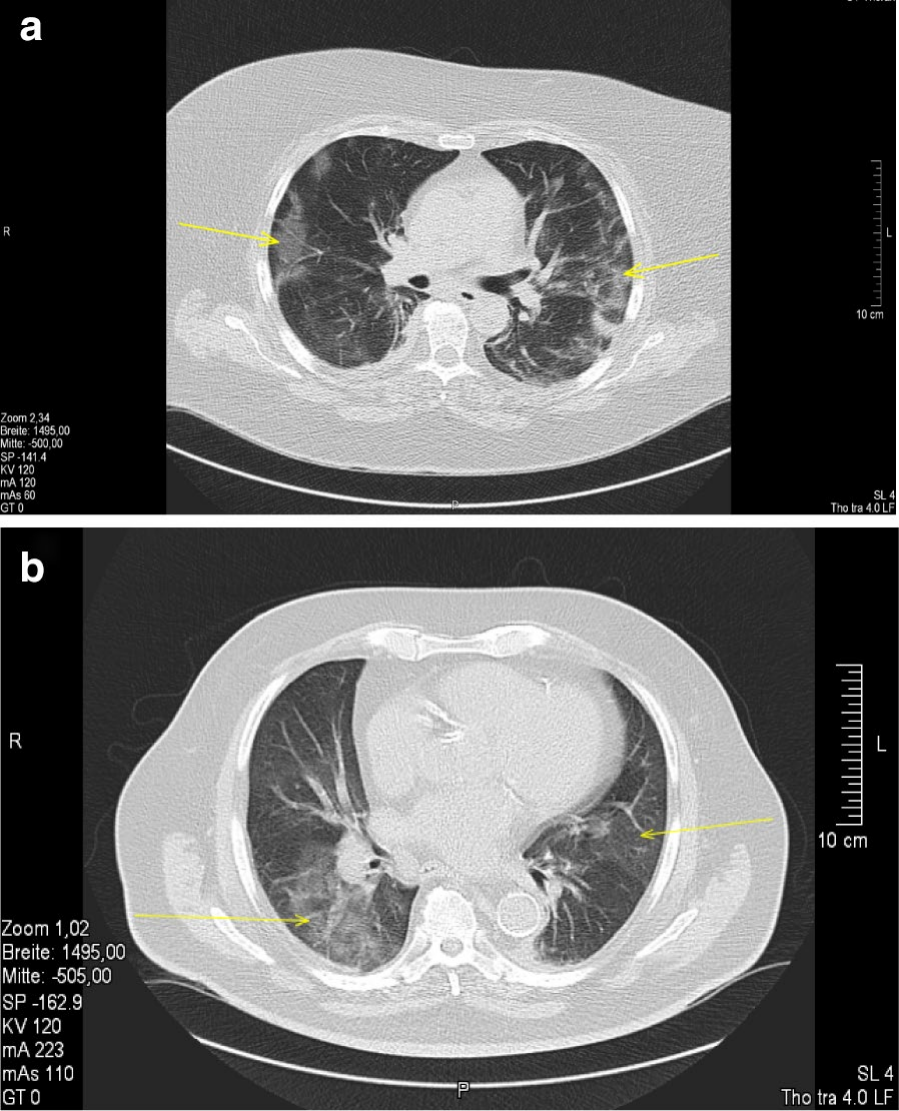
Thoracic computed tomography showing bilateral ground-glass opacities (arrows) of patient 1 (**a**) and patient 2 (**b**)

*Case 1* A 60-year-old Caucasian female patient was admitted to the hospital in March 2020 for suspected pneumonia. She had a history of hypertension and was otherwise healthy. Her oral medication contained an angiotensin receptor blocker (candesartan cilexetil 8 mg twice daily). She had no history of smoking, but did occasionally consume alcohol. She was working as a secretary in a company specialized in processed food supply. She had lived for the last 17 years with her husband in an urban area in the vicinity of our hospital. She reported fever, shivers and cough for 7 days and a change in her sense of taste. The probable time of infection was 8 days prior to the onset of symptoms, when she was in an airport in Austria with many people who were leaving the area after ski vacations. Two days prior to admission, she was given an oropharyngeal and nasopharyngeal swab test by her general physician, which was negative for SARS-CoV-2. The swab test was repeated in our hospital after her admission with a confirmation of the negative result. RT-PCR was conducted according to the TIB MOLBIOL cycling parameters on a Roche LightCycler 480 [1]. At this time, her body temperature was 37.4 °C, blood pressure 128/78 mmHg and heart frequency 61 beats per minute. The physical and neurologic examination was unremarkable, except for her taste loss. Computed tomography showed ground-glass opacities with the beginning of consolidation (Fig. 1a). Bronchoscopy was performed to obtain bronchoalveolar lavage (BAL) fluid, which finally tested positive for SARS-CoV-2. She was provided with subcutaneous antithrombotic prophylaxis (enoxaparin 40 mg once daily). Her laboratory findings are reported in Table 1. Aerobic and anaerobic blood cultures remained sterile, and no fungi were detected.

**Table 1:** Laboratory findings on admission discharge

	Case 1 Admission	Case 1 Discharge	Case 2 Admission	Case 2 Discharge
Creatinine (mg/dl)	2.6	1.6	1.6	1.3
MDRD-GFR (ml/min)	27	48	44	56
Urea (mg/dl)	66	51	91	45
Uremic acid (mg/dl)	7.7	3.4	12.2	7.2
Potassium (mmol/l)	4.3	3.9	5.4	4.1
Sodium (mmol/l)	128	142	127	144
Calcium (mmol/l)	2.36	2.19	2.20	2.21
Bilirubin (mg/dl)	0.3	0.4	0.7	0.5
Aspartate transaminase (IU/l)	110	84	23	27
Glutamic-pyruvic transaminase (IU/l)	64	100	12	30
Gamma-glutamyl transferase (IU/l)	45	50	30	34
Lactate dehydrogenase (IU/l)	494	375	237	220
Alkaline phosphatase (IU/l)	80	74	53	
C-reactive protein (mg/dl)	17.9	1.4	6.5	2.9
Leukocyte (10^3^/µl)	6.4	6.9	19.0	11.4
Hemoglobin (g/dl)	13.6	11.7	11.8	11.0
Erythrocyte (10^6^/µl)	5.15	4.4	3.8	3.7
Hematocrit (%)	40.0	35.6	35.1	34.7
mean corpuscular volume (fl)	78	81	93	95
Mean corpuscular hematocrit (pg)	26	35.6	31	30
Mean corpuscular hemoglobin concentration (g/dl)	34	33	34	32
Red blood cell distribution width (%)	14.3	15.1	13.3	13.1
Thrombocytes (10^3^/µl)	177	267	212	296
Mean platelet volume (fl)	11	11	10	10
Neutrophils (%)	84.3	77.1	70	78.9
Lymphocytes (%)	5.3	9.4	17.2	11.5
Monocytes (%)	10.2	12.4	11.3	7.5
Eosinophils (%)	0.0	0.6	1.1	1.5
Basophils (%)	0.2	0.5	0.4	0.6
Urine proteinuria (dipstick; mg/dl)	25	–	100	100
Urine erythrocyturia (dipstick; per µl)	25	–	250	50
Urine glucosuria (dipstick; mg/dl)	300	–	0	0

*MDRD* Modification of diet in renal disease,* GFR* Glomerular filtration rate

*Case 2* A 83-year-old Caucasian male presented to our hospital for a red swollen lower leg. Moreover, he reported having fever and a mild cough for 4 days. He had a history of diabetes with peripheral artery disease, coronary heart disease and hypertension. The antidiabetic oral medication consisted of metformin (1000 mg twice daily), empagliflozin (10 mg once daily), combined with valsartan (160 mg once daily), amlodipine (10 mg once daily), acetylsalicylic acid (100 mg once daily) and atorvastatin (40 mg once daily). A widower and former employee of a large brewery, he had moved to a retirement home 4 years ago. He had no recent travel history. On physical examination he showed a one-sided diabetic foot syndrome with erysipelas, presenting as a sharply demarcated, hyperthermic and swollen erythema. His temperature was 38.9 °C and blood pressure 134/88 mmHg with a heart frequency of 94 beats per minute. Pulmonary auscultation and the remaining physical examination were unremarkable. No neurologic deficit was detected. Laboratory findings showed a C-reactive protein value of 6.5 mg/dl, lactate dehydrogenase of 237 IU/l and elevated leukocyte count (19.0/nl). Blood cultures did not reveal any pathogens.

In addition to antithrombotic prophylaxis with enoxaparin (40 mg once daily), antibiotic treatment with ampicillin/sulbactam was initiated for erysipelas. His home medication was continued during his hospital stay. Due to the reported cough, a pharyngeal swab test was performed, which was positive for SARS-CoV2. Cough and fever disappeared within the first day after admission, and the erysipelas and general condition gradually improved. At day 5, the pharyngeal swab test was repeated and was negative. A final swab test was scheduled for the next day, and discharge was prepared in case of a negative finding. At this time, however, the patient rapidly developed hypoxia. Computed tomography showed bilateral ground-glass opacities (Fig. 1b), and a BAL was obtained. Both the final pharyngeal swab test and the BAL fluid were negative for 2019 novel coronavirus. Microbiologic examination of the BAL did not detect any coinfection either. Besides a slight lymphopenia (1063 cells/µl) and moderate NK cytopenia (26.4 cells/µl), the cellular immune profiling performed at detection of pneumonia revealed normal absolute and relative numbers of CD4+ and CD8+T cells, B cells, monocytes (including intermediate, classical and non-classical monocytes) and NKT cells. The patient required oxygen supply, but did not need intensive care support. He recovered gradually from the SARS-CoV-2 pneumonia and was discharged 15 days after admission. Both patients had an ambulatory follow-up after 6 months and had recovered completely from their COVID-19-associated symptoms.

## Discussion

We present two cases of COVID-19 pneumonia, which emphasize the aspect of time-dependent SARS-CoV-2 diagnostics. The first case presented with a negative nasopharyngeal swab test by RT-PCR because of viral clearing in the upper respiratory tract. Only bronchoscopy was able to provide the definite diagnosis of COVID-19. The second patient remained RT-PCR negative, even in the lower respiratory tract. Both patients presented with similar and typical radiomorphologic changes in their computed tomography of the chest.

Negative pharyngeal swab tests during SARS-CoV-2 pneumonia have been described before in five Chinese patients [2]. In these cases, however, swab tests became positive in the course of treatment. Our second case reveals that SARS-CoV-2 pneumonia can occur after pharyngeal clearing of the virus with an initially positive and afterwards persistently negative PCR from pharyngeal swab test material. Moreover, the case illustrates that even BAL fluid can be negative for the virus in PCR. The case thereby illustrates an important diagnostic dilemma in patients who present in the later course of infection. Indeed, 100% false-negative PCR results are seen on the day of infection, which decreases down to 38% when clinical signs of COVID-19 appear[3]. SARS-CoV-2 RT-PCR from pharyngeal swab specimens has a high sensitivity in the first week of symptomatic disease in contrast to the following weeks [4, 5]. A pharyngeal PCR can become negative, although the virus persists in the lung. This phenomenon has been described in clinically mild cases of SARS-CoV-2 infections [6]. In the present two cases of 2019 coronavirus disease (COVID-19), however, the virus was not detectable in the pharynx despite massive pulmonary opacities. In the second case, it was not even detectable in BAL. This finding may be explained by the time course of COVID-19. In the first week, the virus predominantly replicates in the epithelia of the upper airways and causes cold-like symptoms such as a sore throat, cough and potentially elevated body temperature [7]. A subsequent replication in the lower airways initiates an inflammatory response, which becomes radiologically visible as pulmonary infiltration. The present case illustrates that inflammation may be perpetuated although the virus has been cleared.

This finding is of substantial clinical relevance. Many patients do not seek health care during the early period of infection as they only have mild upper airway symptoms. Moreover, nasopharyngeal swab tests are highly operator dependent. Pneumonia, however, occurs a median of 5–8 days after infection [7, 8]. The present findings show that both a negative pharyngeal swab test and a negative BAL do not rule out COVID-19 at this time even though pulmonary infiltration may be significant. Therefore, the indication for computed tomography should be considered liberally. In case of typical imaging findings, specimens from the lower respiratory tract such as the sputum, endotracheal aspirate or BAL should be obtained. Even in case of a negative finding, COVID-19 has to be considered as a differential diagnosis. Otherwise, these patients' symptoms may be misinterpreted as pneumonia of another origin.

In summary, negative RT-PCR findings from pharyngeal swab tests and even lower respiratory specimens can be negative in SARS-CoV-2 pneumonia. Computed tomography is the crucial diagnostic prerequisite to avoid overlooking SARS-CoV-2 infection in these patients.
